# Genetic Scores of *eNOS*, *ACE* and *VEGFA* Genes Are Predictive of Endothelial Dysfunction Associated Osteoporosis in Postmenopausal Women

**DOI:** 10.3390/ijerph18030972

**Published:** 2021-01-22

**Authors:** Puneetpal Singh, Monica Singh, Rubanpal Khinda, Srishti Valecha, Nitin Kumar, Surinderpal Singh, Pawan K. Juneja, Taranpal Kaur, Sarabjit Mastana

**Affiliations:** 1 Division of Molecular Genetics, Department of Human Genetics, Punjabi University, Patiala 147002, India; singhmonica2017@gmail.com (M.S.); ruban_rs19@pbi.ac.in (R.K.); srishti_rs19@pbi.ac.in (S.V.); Nitin_rs19@pbi.ac.in (N.K.); 2 Aggarwal Orthopedic Hospital, Ludhiana 141001, India; spsaoh@gmail.com (S.S.); pwkjaoh2016@gmail.com (P.K.J.); 3 Amrit Sagar Hospital, Ferozepur 152001, India; tpashf2012@gmail.com; 4 Human Genomics Lab., School of Sport, Exercise and Health Sciences, Loughborough University, Loughborough LE11 3TU, UK

**Keywords:** predictive models, endothelial dysfunction, osteoporosis, bone mineral density, SNP-SNP interactions, genetic models, haplotypes

## Abstract

The present study aimed to examine the participation and contribution of endothelial nitric oxide synthase (*eNOS*), angiotensin converting enzyme (*ACE*) and vascular endothelial growth factor (*VEGFA*) genes for the risk of endothelial dysfunction (ED)-associated osteoporosis risk in postmenopausal women of Punjab, India. Women with ED were categorized into women with osteoporosis (n = 346) and women without osteoporosis (n = 330). They were examined for selected SNPs within *eNOS, ACE* and *VEGFA* genes. Linear regression analysis revealed a positive association of ED with bone mineral densities (BMDs) at femoral neck (r^2^ = 0.78, *p* < 0.001) and lumbar spine (r^2^ = 0.24, *p* = 0.001) after Bonferroni correction. Three susceptibility haplotypes were exposed within *eNOS* (CTAAAT), *ACE* (ACDG) and *VEGFA* (GATA) genes. Bearers of CTAAAT (OR 2.43, *p* = 0.007), ACDG (OR 2.50, *p* = 0.002) and GATA (OR 2.10, *p* = 0.009) had substantial impact for osteoporosis after correcting the effects with traditional risk factors (TRD).With uncertainty measure (*R*^2^_h_) and Akaike information criterion (AIC), best fit models showed that CTAAAT manifested in multiplicative mode (β ± SE: 2.19 ± 0.86, *p* < 0.001), whereas ACDG (β ± SE: 1.73 ± 0.54, *p* = 0.001) and GATA (β ± SE: 3.07 ± 0.81, *p* < 0.001) expressed in dominant modes. Area under receiver operating characteristic curve using weighted risk scores (effect estimates) showed substantial strength for model comprising TRD + GATA (AUC = 0.8, *p* < 0.001) whereas, model comprising TRD + GATA + CTAAAT exhibited excellent ability to predict osteoporosis (AUC = 0.824, *p* < 0.001)

## 1. Introduction

Osteoporosis is a complicated skeletal disorder, which is influenced by multiple factors and influences multiple elements of health, especially in women after menopause owing to endocrinological, physiological and psychological upheaval. Primarily, it is confirmed by low bone mineral density (BMD) that leads to brittle bones and fractures. The scientific literature reveals that during this phase of life, reduced hormone levels severely affect the vascular endothelium, leading to endothelial dysfunction [1,2,3]. Endothelial dysfunction is an abnormal state, whereby the endothelial cell walls fail to balance between vasorelaxation and vasoconstriction, due to reduced synthesis and availability of nitric oxide (NO), which largely mediates, manages and maintains it. Post-menopause-associated low estrogen levels downregulate NO production, resulting in impaired vascular tone and reactivity [4,5]. Estrogen receptors present within the endothelium are activated by estrogen concentrations and consequently augment the release of NO [6]. Hormone therapy improves vascular function by reducing pro-inflammatory, pro-oxidative and pro-thrombotic states [7], which is further corroborated by the finding that estrogen-induced release of nitric oxide synthase can be blocked by specific estrogen antagonists [8].

Deficiency of hormone levels after menopause severely increases bone resorption and the bone formation process is slowed down [2,4]. Bone loss during bone resorption is compensated by replacing osteoclasts (bone resorbing cells) with osteoblasts (bone forming cells) on the bone surface. Osteoblasts initially form an immature and unmineralized bone-like matrix called the osteoid. Endothelial dysfunction obstructs the flow of blood that is rich in oxygen, nutrients and metabolites being supplied to the osteoid hence hindering its calcification and mineralization [9], while continuous and sufficient blood supply to the bone enhances bone mineralization, its repair and maintenance [9,10]. This is consistent with the finding that women suffering coronary endothelial dysfunction are at higher risk of developing osteoporosis than their normal counterparts [1]. Furthermore, substantial supply of blood to the bone reassures bone remodeling [11], creates a suitable microenvironment for growth factors and metabolites in fractured bone [9], reduces inflammation for bone healing [12] and couples osteogenesis and angiogenesis in bone homeostasis [13]. Alluding to these findings, it is evident that a clinically viable link exists between hormone levels, osteoporosis and endothelial dysfunction. Clinical reports have recognized that hormonal deficiency regulates endothelial dysfunction [14,15], but whether such a link is detrimental to bone mass has largely been undefined so far except for a few findings [16,17].

Besides other therapeutic modalities of osteoporosis, hormone replacement therapy (HRT) is efficient, however, it correlates significantly with a higher breast cancer risk. Estrogen-related receptor alpha (ERR-α), an orphan receptor, augments cell proliferation and tumorigenesis in HRT-mediated breast cancer [18]. Moreover, its expression is inversely associated with attenuated osteoblast differentiation hence, reduced BMD. It has been observed that cholesterol and mevalonate are associated with the initiation, progression and aggressiveness of breast cancer risk which are transcriptionally regulated by ERR-α in ER+ and triple negative breast cancer (TNBC) cells [18,19]. Consequently, ERR-α has been suggested to be an important target for osteoporosis treatment, however its controversial role in up-regulation of NO cannot be ignored [20].

Biosynthesis of NO is catalysed by oxidation of L-arginine to L-citrulline, which is controlled by the transcriptional action of the endothelial nitric oxide synthase (*eNOS*) gene. It is 21 kb gene that contains 26 exons and mapped to 7q35–36. Amongst several polymorphisms within and around this gene, single nucleotide polymorphism (SNP); rs2070744 (−786T/C) has been observed to be functional and have significant influence on NO production [17].

Angiotensin converting enzyme (*ACE*) is the key player in inactivating bradykinin and converting angiotensin I to vasoconstrictor angiotensin II by transcriptional regulation of *ACE* gene that possesses 26 exons and localized on 17q23.3. An insertion-deletion polymorphism within the *ACE* gene (rs1799752) has been confirmed to regulate half of the serum *ACE* levels [21]. This polymorphism impacts endothelial dysfunction by augmenting angiotensin II-induced catalysis of nitric oxide (NO) and attenuates bradykinin regulated NO release [21].

Vascular endothelial growth factor (*VEGF*), a signaling protein is a growth factor which promotes endothelial cell growth, angiogenesis and vasculogenesis [22]. Insufficiency of *VEGF* is associated with strong endothelial dysfunction via attenuation of NO release whereas administration of *VEGF* repairs endothelial cell injury [23]. These functions are observed to be controlled by *VEGFA* gene expression [24]. Within bone, hypoxia-inducible factor (HIF-I) upregulates osteoblast-mediated VEGF expression, balances between angiogenesis and osteogenesis and promotes bone formation [23,24], whereas reduced VEGF expression prompts reduced osteoblast differentiation thereby promoting osteoporosis-like phenotypes [24]. The major allele G of a functional SNP rs2010963 (−1154G/C) of *VEGFA* gene has been observed to be associated with reduced *VEGF* transcription [25].

Many reports have analysed independently the role and contribution of these three pertinent candidates—*eNOS, ACE* and *VEGFA* genes—in vascular function [17,21,25]. These genes regulate endothelial function in various systems of the body through different mechanisms, especially in cardiovascular pathology, but their participation in bone vasculature remains elusive. Apropos to this, the present study aims to investigate the genetic contribution of some important SNPs of these genes through their gene-gene, gene-environment and haplotype specific interactions as genetic determinants and predictors of endothelial dysfunction inflicted osteoporosis in postmenopausal women. 

## 2. Subjects and Methods

### 2.1. Subjects

**Figure 1:**
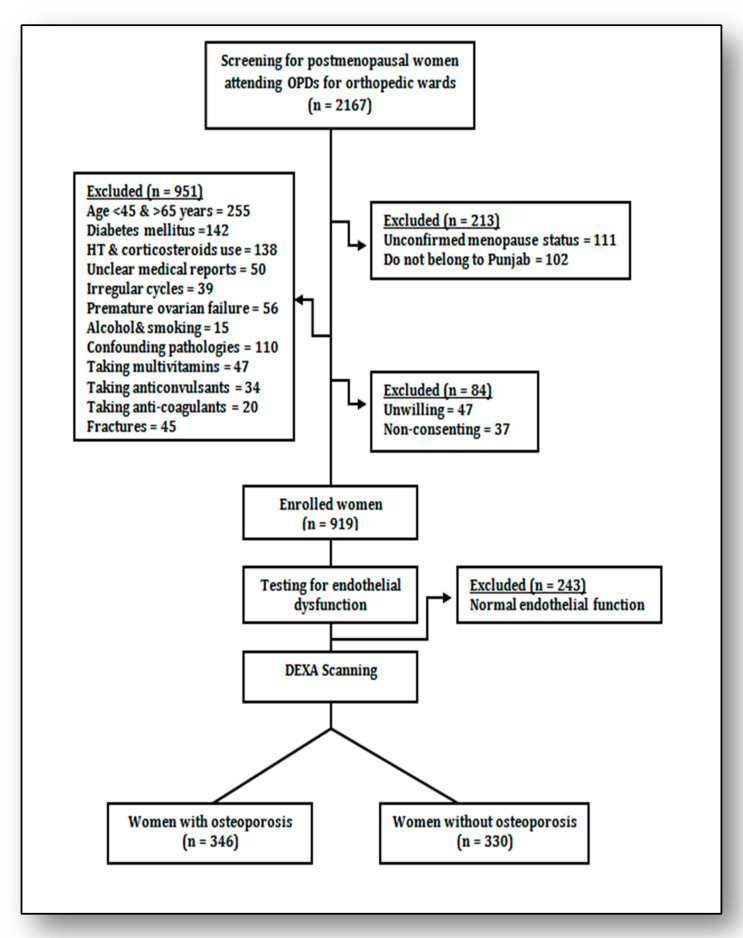
Showing data collection protocol.

In the present study, preliminary examination of 2167 postmenopausal women who visited orthopedic wards of prominent hospitals (Rajindra Hospital, Aggarwal Orthopedic Hospital and Amrit Sagar Hospital) of Punjab, India was conducted to establish their baseline characteristics. After following the exclusion and inclusion criteria, 919 women were enrolled (Figure 1). Their endothelial damage was tested and reactive hyperemia index (RHI), an indicator of endothelial dysfunction, was assessed (EndoPAT 2000 device by Itamar Medical Technology Ltd., Caesarea, Israel). After exclusion of 243 women who had normal vascular function, 676 postmenopausal women finally participated. Their BMD was examined (dual energy x ray absorptiometry: DXA) at L1 to L4 vertebrae of lumbar area (BMD_LS) and at neck of the femur (BMD_FN). Based on criteria of T scores given by WHO, 346 women had scores ≤ 2.5 and were categorized as women with osteoporosis. The remaining 330 women had T scores ≥ 1 and hence were categorized as women without osteoporosis. Considering aim of the study which demands clear pathologies and minimum stratification, so as to deduce exclusive effect of endothelial dysfunction, women having T-score between −1.0 and −2.5 (osteopenia) and women having fractures were not included. It is reasonable to investigate effect of endothelial dysfunction on confirmed low BMD (osteoporosis) whereas, osteopenia is midway phenotype where the effects of low bone mass has just started to express. Similarly, fractures may confound the analysis because these can be the outcomes of bone cysts, cancers or excessive use of glucocorticoids and bisphosphonates, irrespective of low BMD.

Only those subjects who had given their informed written consent were allowed to participate in the study. In order to avoid any bias, the case control status was coded and blinded to the researchers. The protocol of the study was approved by institutional ethical review board (Reference no. IEC2017/05, dated 20 January 2017) and conformed strictly to ethics for medical research involving human subjects (Helsinki Declaration).

### 2.2. Description of Variables

Body mass index (BMI) was calculated by Quetelet’s index, which is weight in kilograms over height in meters squared (kg/m^2^). Detailed menopause status for recording age and years since menopause (YSM) were verified from their medical records or through personal interviews. Systolic (SBP) and diastolic blood pressure (DBP) measurements were conducted, two times on resting subjects (at least for 10 min) after an interval of 3 min and their mean values were recorded. Lipid values of triglycerides (TG), high density lipoproteins (HDL) and total cholesterol (TC) were analysed by using assay kits (Erba Mannheim, London, UK) with one step enzymatic methods on Lisa scan plate reader (Erba Mannheim). It can identify a minimum of 0.1 mg/L of component. Low density lipoproteins (LDL) levels were calculated with the Friedewald equation. The inter-assay and intra-assay coefficients of variation (CVs) were 6.2 and 6.7, respectively.

### 2.3. Examination of Endothelial Function

A non-invasive technique using an EndoPAT 2000 device was conducted to check reactive hyperemia index (RHI), an indicator of endothelial function. According to the manufacturer’s guidelines patients were counseled to not take any food items or beverages containing methylxanthine (caffeine, tea, chocolate, etc.,) at least 5 h before examination. After taking mean blood pressure of two measurements, finger probes were positioned, which measured endothelium dependent change in vascular tone. First of all baseline pulse variation was noticed and recorded for about six minutes and then occlusion to brachial artery was done up to at least 250 mmHg of systolic blood pressure with sphygmomanometer cuff. Non-endothelium-dependent alterations of the contra-lateral arm were compared to assess vascular tone. Now occlusion was released after 5 min which causes flow mediated dilation (FMD) and EndoPAT recorded RHI values as increase in pulse amplitude tone (PAT). Subjects having RHI < 1.67 were confirmed to have endothelial dysfunction. These measurements were operator independent being accomplished by in-built algorithm based dedicated software.

### 2.4. BMD Evaluation

DXA was employed to test BMD by using Hologic QDR 4500 system (Hologic Inc. Waltham, MA, USA). T scores were inferred based on their comparisons with peak bone mass of average 30 years old young individual of the same gender. On the basis of these T scores, those women were confirmed to have osteoporosis who had T score ≤ 2.5 and women without osteoporosis; who had T scores ≥ 1. The QDR system was standardized according to the guidelines of the manufacturer before testing. Inter- and intra-assay CVs for the checking of the BMD_FN and BMD_LS were 5.0 and 5.8, respectively.

### 2.5. Selection of the SNPs and Genotyping

In the clinical arena, it is well understood that functional manifestations of these three genes (*eNOS, ACE* and *VEGFA*) strongly impact endothelial function by inducing NO bioavailability in the endothelium, generation of super oxide anions that degrade NO and induce angiogenesis [17,21,22,23,24,25]. Functional SNPs along with other significant candidates for each gene were selected on the basis of three points; (i) these SNPs should have been earlier verified by submitted information on the reference SNP collection databank at dbSNP (http://www.ncbi.nlm.nih.gov/snp), (ii) these SNPs should have been formerly identified as having an association with endothelial dysfunction (iii) all the SNPs should have been polymorphic with minor allele frequency > 0.05. Following these criteria, six SNPs of *eNOS* gene i.e., rs2070744, rs1799983, rs1800780, rs3918181, rs891512 and rs1808593, four SNPs of *ACE* i.e., rs4459609, rs1800764, rs1799752 and rs4343 and four SNPs of *VEGFA* i.e., rs2010963, rs699947, rs833061 and rs1570360 were selected. After extraction of deoxyribose nucleic acid (DNA) from whole blood with a normal salting-out procedure, PCR was used to amplify DNA with reaction mixture of 25 μL. High conformity restriction enzymes (NEBS, Hertfordshire, UK) were used to digest amplicons. Depending upon the product size, genotypes were assessed and typed on two percent to three percent agarose gels. The confidentiality of the subjects was maintained and all the experimental work was blinded to the clinical and case-control status in order to avoid any subjective bias. To verify reproducibility of genotyping, 15 percent of the respective samples were re-analyzed.

### 2.6. Population Stratification Analysis and Statistical Power of Genetic Association

Case-control design of the study may assume false positive inferences because of underlying population stratification (PS), which shows misleading differences in allele frequencies due to different ancestries, otherwise considering subpopulations of the population belonging to same ancestry. Using the software Arlequin ver. 3.0 [26], population comparisons were performed to compute pairwise fixation index (F_st_), an indicator of how populations differ genetically. F_st_ for within population differentiation was observed to be 0.035 ± 0.019, which showed that no considerable PS existed between groups in this study population that may confound the genetic analysis. Genetic association versus sample size was examined with 676 subjects (346 cases and 330 controls) by using the Power for Genetic Association Analysis (PGA) Package [27], which deduced appropriate sample size and minimum detectable relative risk (MDRR) using SNPs and haplotype effects under various models. A preliminary analysis indicated that this sample size (n = 676) would deliver more than 90 percent power to differentiate minimum genotype relative risk (MGRR) of 2.0 with apportioned value of minor allele frequency of at least 0.21 (rs1799883 in present sample) at significance of 0.05. Analysis with haplotype effect module suggested that sample size of 676 would deliver MDRR of 1.5 with substantial power (>90%). Further, power of genetic association was checked at more stringent significance levels (0.01, 0.001), which suggested that this sample size is sufficient to discriminate between genotype relative risk of 2.0 under all the genetic models (additive, dominant and recessive) with more than 80 percent statistical power.

### 2.7. Statistical Analysis

To examine differences between proportions or categorical data of the study groups, a chi-square test was applied, whereas a t test (Student’s t) or Mann-Whitney-Wilcoxon rank-sum test was used for continuous data. Gene counting was done for calculating minor allele frequencies and Hardy-Weinberg equilibrium was evaluated with Fischer’s exact test. Inter-relationship of BMD (target variable) at both sites and endothelial dysfunction (predictor variable) was assessed with linear regression analysis and summarized in whisker plots. Variance inflation factor (VIF) was calculated to check collinearity between both the explanatory variables (BMD_FN and BMD_LS). Extent of association between all the variables with BMD was examined with univariate regression analysis (GLM procedure). Those variables which showed significance in univariate model were involved in binary logistic regression analysis (backward step method) to identify their independent relationship with the dependent variable along with interaction analyses between them. Full logistic regression analysis was applied to calculate association of designated alleles with the risk of endothelial dysfunction associated osteoporosis in codominant, dominant, recessive, and multiplicative genetic models. Assuming genetic disease penetrance of 1, r and r^2^ for genotypes AA, AB and BB respectively, codominant model specifies that risk of osteoporosis is increased by r-fold for genotype AB and r^2^-fold for genotype BB. For dominant model, either one or two copies of allele B are required for r-fold increased risk, recessive model demonstrates that two copies of allele B are required for r-fold increased risk and multiplicative model indicates that the osteoporosis risk is increased by r-fold with each additional *B* allele. Two locus epistasis effect between SNPs versus risk variables (gene-environment relationship) were analysed using the epiSNP software [28]. Haplotypes were generated using genotype data with the software Arlequin ver. 3.0 [26]. In order to understand risky haplotypes, multivariable regression analysis was used to compute odds ratios in unadjusted model and a model with adjusted values for risk variables after taking most prevalent haplotype as referent. Risky haplotypes were checked more stringently to understand their best modes of impact in different genetic models (Dominant, Recessive, Multiplicative and general). Best fit model was identified with Wald test and Akaike information criterion (AIC = −2 log-likelihood + 2 × number of parameters). Degree of haplotype uncertainty (*R*^2^h) was also investigated by the method of Stram et al. [29].

### 2.8. Receiver Operating Characteristic Curve Analysis

For the evaluation of discriminatory ability and predictive accuracy of collective effects of alleles within a gene (haplotype) and/or traditional risk factors (BMI, SBP and TG), area under receiver operating characteristic curve (AUROC) was modeled with risk scores. Risk scores for traditional risk factors (TRD) were taken as respective β coefficients (unweighted scores) obtained from logistic regression analysis. These values were further standardized by multiplying lowest absolute value of the coefficient with a number to become value of 1 and all β values were multiplied by that number to round them to the closest integer (weighted scores). For calculating genetic risk scores of haplotypes, individual scores were calculated based on the carriage of risky alleles in the susceptibility haplotype of each gene. Genotypes of risk (R) and non-risk (N) alleles were apportioned scores of 0 (NN), 1 (RN) and 2 (RR) for each SNP participating in respective susceptibility haplotype. Final risk score was deduced by summing up all SNP wise risk score of every individual. The values of area under curve (AUC), from 0.6- to 0.7, 0.7 to 0.8 and 0.8 to 0.9 were considered weak, acceptable and excellent, respectively.

## 3. Result

### 3.1. Analysis of Variables at Baseline and Genetic Correlates

Baseline variables of postmenopausal women having endothelial dysfunction categorized according to osteoporosis status are summarized in Table 1. No evidence of statistically significant differences between the two groups for age, YSM, DBP, LDL, HDL, and TC (*p* > 0.05) was observed. Values of BMI, SBP and TG were considerable higher in women having osteoporosis than women without osteoporosis and these differences were statistically significant (*p* < 0.001) between both the groups. Albeit, all the postmenopausal women participated in the present study had confirmed endothelial dysfunction (RHI < 1.67), nonetheless, its effect was considerably enlarged in women having osteoporosis and differences between the groups were statistically dissimilar (*p* < 0.001). Similarly, it is evident that adjusted values of BMD at femoral neck and lumbar spine were markedly low in women having osteoporosis than women without it (*p* < 0.00).

Genotype frequencies of all the SNPs of three genes (*eNOS, ACE* and *VEGFA*) were within range (*p* > 0.05) of Hardy-Weinberg equilibrium. Minor allele frequencies (MAF) of rs1800780, rs3918181 and rs1808593 of eNOS gene were similar between both groups (*p* > 0.05), however, for rs2070744, rs1799983, and rs891512, MAF differed significantly (*p* < 0.05) between osteoporotic and non-osteoporotic women. MAF of three SNPs of ACE gene i.e., rs1800764, rs1799752 and rs4343 were observed to be significantly dissimilar (*p* < 0.05), however MAF of rs4459609 was similar (*p* = 7.86) between women having osteoporosis and women without it. MAFs of SNP rs833061 within VEGFA gene were detected to be non-significant (*p* = 0.793) between both the groups of women, whereas MAFs of other three SNPs; rs2010963, rs699947 and rs1570360 were observed to be considerably different (*p* < 0.05) between them.

### 3.2. Identification of Independent Risk Variables

Univariate analysis of risk variables were analysed for assessing their impact on osteoporosis (Table 2), which showed that BMI, YSM, SBP, TG and BMDs at both the sites of femoral neck and lumbar spine were significant risk variables. Further, their analysis in binary logistic regression analysis showed that BMI (≥30 kg/m^2^), YSM (>5 years), SBP (>120 mmHg), TG (>150 mg/dL) and BMDs at femoral neck (<0.7 g/cm^2^) and lumbar spine (<0.8 g/cm^2^) were independent risk predictors (*p* < 0.05) for osteoporosis risk, whereas, DBP, TC, LDL and HDL did not influence osteoporosis risk (*p* > 0.05).

### 3.3. Genotype Specific Implications of Genes through Different Genetic Models

Role and relevance of individual SNPs of *eNOS* gene were analysed by comparing carriage of risky allele between women with osteoporosis and women without osteoporosis by taking major allele as referent (Table 3). Genotype specific codominant, dominant, recessive and multiplicative models after adjusting the effect of variables revealed that minor allele C of rs2070744 were associated in codominant in heterozygous (OR 1.55, 95%CI: 1.18–2.19, *p* = 0.007), homozygous (OR 1.73, 95%CI: 1.09–3.82, *p* = 0.043), dominant (OR 1.65, 95%CI: 1.10–3.19, *p* = 0.037) and multiplicative modes (OR 1.33, 95%CI: 1.14–2.00, *p* = 0.005). Similarly, minor alleles T and A of rs1799983 and rs891512 respectively showed association in codominant in heterozygous (ORs 2.53 & 1.55, 95%CIs: 1.81–3.91, 1.15–2.28, *p* = 0.006 &0.029), homozygous (ORs 3.00 & 2.20, 95%CIs: 1.14–8.19, 1.10–4.92, *p* = 0.042 & 0.040), dominant (ORs 2.84 & 1.67, 95%CI: 1.66–5.29, 1.02–2.77, *p* = 0.009 & 0.050) and multiplicative modes (ORs 2.13 & 1.51, 95%CI: 1.27–2.92, 1.13–2.09, *p* = 0.002 & 0.034). SNPs rs1800780, rs3918181 and rs1808593 did not associate with osteoporosis risk.

SNP rs4459609 polymorphism of *ACE* gene failed to show any association with osteoporosis risk (Table 4) whereas, minor allele C of rs1800764 showed association in codominant homozygous (OR 1.71, 95%CI: 1.08–3.17, *p* = 0.038), and multiplicative modes (OR 1.22, 95%CI: 1.01–1.62, *p* = 0.043). This allele could not retain its significance in dominant mode after adjustment with the variables (*p* > 0.05). Another in/del polymorphism (rs1799752) of *ACE* gene showed strong association of D allele in all the possible modes i.e., codominant in heterozygous (OR 1.37, 95%CI: 1.05–2.27, *p* = 0.044), homozygous (OR 2.19, 95%CI: 1.33–3.41, *p* = 0.007), dominant (OR 1.68, 95%CI: 1.20–2.38, *p* = 0.013), recessive (OR 1.42, 95%CI 1.03–2.13, *p* = 0.028) and multiplicative (OR 1.22, 95%CI: 1.11–1.69, *p* = 0.008). Another SNP rs4343 showed that the minor allele G was associated in codominant in heterozygous (OR 1.51, 95%CI: 1.14–2.17, *p* = 0.009), dominant (OR 1.43, 95%CI: 1.17–2.00, *p* = 0.028) and multiplicative modes (OR 1.29, 95%CI: 1.09–1.72, *p* = 0.015).

Functional SNP rs2010963 of *VEGFA* exhibited significant protective association in codominant in heterozygous (OR 0.60, 95%CI: 0.42–0.83, *p* = 0.027), dominant (OR 0.57, 95%CI: 0.41–0.79, *p* = 0.036) and multiplicative (OR 0.65, 95%CI: 0.50–0.82, *p* = 0.010) modes (Table 5). Minor allele C of SNP rs699947 was observed to be associated in codominant in homozygous (OR 1.72, 95%CI: 1.11–2.72, *p* = 0.027), dominant (OR 1.30, 95%CI: 1.00–1.82, *p* = 0.048), recessive (OR 1.51, 95%CI: 1.01–2.24, *p* = 0.039) and multiplicative modes (OR 1.22, 95%CI: 1.05–1.61, *p* = 0.027). Minor allele A of another SNP rs1570360 was observed to be associated with osteoporosis risk in codominance in heterozygous (OR 1.52, 95%CI: 1.11–2.19, *p* = 0.011), homozygous (OR 2.13, 95%CI: 1.17–3.29, *p* = 0.026), dominant (OR 1.69, 95%CI: 1.20–2.31, *p* = 0.017) and multiplicative modes (OR 1.53, 95%CI: 1.18–1.95, *p* = 0.025). None of the models showed significant association of SNP rs833061 with osteoporosis risk (*p* > 0.05).

### 3.4. Analysis of Linear Relationship of RHI with BMD

**Figure 2:**
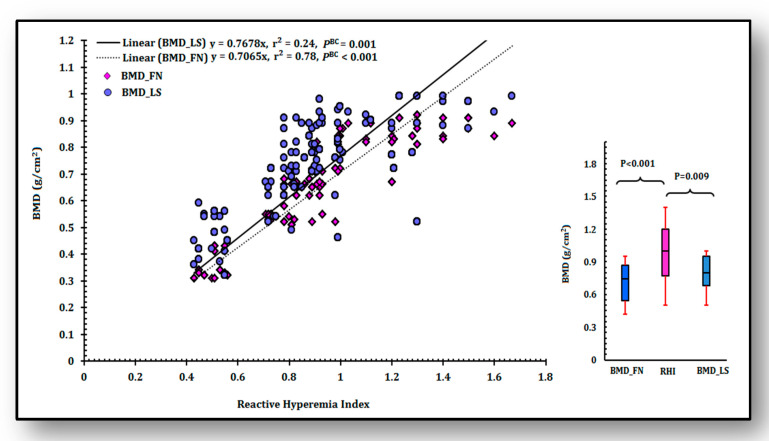
Linear regression analysis between bone mineral density (BMD_FN and BMD_LS) and endothelial dysfunction (Reactive Hyperemia Index). *p* values are corrected with Bonferroni (*P*^BC^) for correlation coefficients (r^2^). Box and whisker plots depict median values along with first interquartile and third interquartile values. BMD_FN: bone mineral density at femoral neck, BMD_LS: bone mineral density at lumbar spine, RHI: reactive hyperemia index.

Linear regression analysis (Figure 2) displayed that RHI predicted the values of BMD linearly at both femoral neck (r^2^ = 0.78, *p* < 0.001) and lumbar spine (r^2^ = 0.24, *p* = 0.001) after Bonferroni correction. Results in the analysis indicated that RHI and BMD were positively correlated and that with gradual increase of RHI (normal endothelial function, otherwise lesser the RHI < 1.67, more is the severity of endothelial dysfunction), BMDs at both sites also increased.

In multicollinearity analysis, VIF was observed to be 1.3, which suggested that BMDs at femoral neck and lumbar spine were not linearly dependent on each other. Whisker plots were generated after omitting outliers to clarify this relationship of RHI and BMD, which reassured that RHI positively associated with BMD at femoral neck (*p* < 0.001) and lumbar spine (*p* = 0.009).

### 3.5. SNP-SNP Cross Talks, Risky Traits and Their Modes of Association

Several genotype specific single marker effects within SNPs of *eNOS, ACE* and *VEGFA* genes on risk covariates were deduced. All significant single marker effects (*p* < 0.05, r > 0.04) were further analysed for pair wise epistatic effects with Bonferroni corrections (Table 6). It was revealed that 10 SNP pair associations with risky traits were evident of osteoporosis risk. Functional SNP of eNOS gene; rs2070744 showed epistatic relationship with SNPs rs4343, rs1799983, rs1800764 and rs891512 influencing RHI (*p* = 0.001), LDL (*p* = 0.005), TG (0.001) and SBP (*p* = 0.003) through interactive (I), additive x additive (AA), dominant × additive (DA) and additive × dominant (AD) modes respectively. Interestingly, SNP pairs rs2070744-rs1800764 impacted TG through DA mode in control subjects (*p* = 0.041) also. Another SNP pairs which showed impact on the risk of osteoporosis through AA, dominant × dominant (DD) and I mode were rs4343-rs2010963 (*p* = 0.009), rs1800764-rs1799752 (*p* = 0.029) and rs1799983-rs891512 (*p* = 0.032) influencing RHI, TC and RHI respectively. Similarly, SNP rs1799983 coupled with rs699947 (*p* = 0.001), rs699947 with rs1799752 (*p* = 0.003) and rs891512 with rs1799752 (*p* = 0.022) to influence BMI, RHI and LDL through DD, I and DA modes, respectively. SNP pair rs1799983-rs699947 was observed to afflict DD influence in women without osteoporosis also (*p* = 0.041). Two locus SNP-SNP epistatic links without risk variables have been shown in figure embedded in the Table 6, to have quicker glance without some perplex interactions.

### 3.6. Haplotype Analysis, Their Contribution and Best Mode of Impact for Osteoporosis Risk

SNPs within *eNOS* gene (in the order of rs2070744, rs1799983, rs1800780, rs391881, rs891512 and rs1808593) developed into 64 possible haplotypes and out of them 29 haplotypes were visible. Of these, 21 haplotypes had frequencies less than 5 percent, therefore, excluded from the analysis. The remaining eight haplotypes captured 85percent of genetic variance of women having normal bone mass and 88 percent of women having osteoporosis (Table 7).The major alleles at position 1, 2, 5 and 6 and minor alleles at 3 and 4 of studied *eNOS* SNPs appeared in the form of haplotype TGAAGT was having highest frequency, hence served as referent for the analysis. Minor alleles of all the SNPs except at position 6in the form of CTAAAT appeared to be the risky haplotype for osteoporosis risk (OR 2.80, 95%CI: 1.53–5.13, *p* = 0.001). Inter group comparisons of this haplotype after Bonferroni corrections exhibited significant differences approaching GWAS *p* values (*p* = 1 × 10^−8^). It was observed to be susceptibility haplotype for the risk of osteoporosis (OR 2.43, 95%CI: 1.22–4.71, *p* = 0.007), when its influence was examined after adjusting the effects of confounders (BMI, YSM, SBP and TG).

Haplotype analysis of SNPs in the order of rs4459609, rs1800764, rs1799752 and rs4343 of *ACE* gene exhibited 11 visible haplotypes but six haplotypes were excluded as these had lower frequencies (<5 percent) and were not amenable to be used for authentic results. The remaining five haplotypes showed 88–90 of genetic variability in both the groups of women. All the major alleles representing ATIA haplotype emerged to be the most common haplotype in both the groups, so it was taken as referent. All minor alleles of *ACE* gene SNPs, except at position 1 in the form of haplotype ACDG appeared to be risky (OR 3.03, 95%CI: 1.86–4.88, *p* < 0.001) and it was confirmed that it conferred 2.5 fold higher risk of developing osteoporosis in postmenopausal women having endothelial dysfunction after correcting the effect of risk predictors (OR 2.50, 95%CI: 1.28–3.96, *p* = 0.002).

SNPs of *VEGFA* gene (in the order of rs2010963, rs833061, rs699947 and rs1570360) demonstrated 11 haplotypes with five haplotypes having the least frequencies (<5 percent) hence excluded, leaving six haplotypes that were included for further analysis. Haplotype analysis after taking haplotype GCTG as reference, revealed that GATA is a risky (OR 2.83, 95%CI: 1.65–4.86, *p* < 0.001) whereas CACG is a protective haplotype (OR 0.51, 95%CI: 0.28–0.92, *p* = 0.035) for endothelial dysfunction associated osteoporosis risk. However, CACG could not retain its significance (OR 0.78, 95%CI: 0.45–1.33, *p* = 0.43) whereas, haplotype GATA maintained its significance after adjusting the effects of risk variables (OR 2.10, 95%CI: 1.31–3.29, *p* = 0.009).

Although the results implied that those postmenopausal women who possessed these susceptibility haplotypes were at higher risk of developing osteoporosis than those women who did not have it, in which best possible way these haplotypes inflicted their maximum effects needed to be identified (Table 8). Their functional effects on BMD were modeled and tested with Wald statistics under dominant, recessive, multiplicative and general modes of inheritance and selection of the best fit model was identified with AIC and *R*^2^_h_ (Table 8). Analysis clarified that susceptibility haplotype CTAAAT of eNOS gene influenced osteoporosis risk in multiplicative mode (β ± SE: 2.19 ± 0.86, *p* < 0.001), haplotypes ACDG of ACE gene (β ± SE: 1.73 ± 0.54, *p* = 0.001) and haplotype GATA of VEGFA gene (β ± SE: 3.07 ± 0.81, *p* < 0.001) influenced bone mass in postmenopausal women in dominant modes.

### 3.7. Predictive Ability of Haplotypes and Traditional Risk Factors for the Diagnosis of Osteoporosis

**Figure 3:**
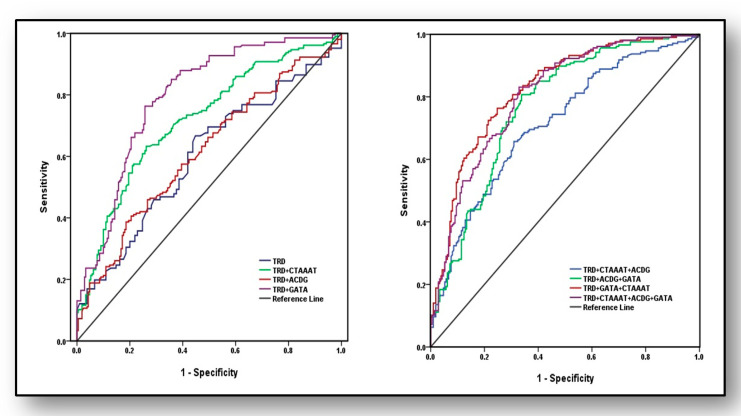
Areas under the receiver operating characteristic (AUROC) curves for the analysis of predictive Scheme 0.028, 95%CI: 0.545–0.654, *p* < 0.001), TRD + CTAAAT (0.72 ± 0.025, 95%CI: 0.670–0.767, *p* < 0.001), TRD + ACDG (AUC 0.614 ± 0.027, 95%CI: 0.560–0.668, *p* < 0.001) and TRD + GATA (AUC 0.80 ± 0.022, 95%CI: 0.751–0.838, *p* < 0.001). In the second set of model, TRD + CTAAAT + ACDG (AUC 0.71 ± 0.025, 95%CI: 0.665–0.762, *p* < 0.001), TRD + ACDG + GATA (AUC 0.77 ± 0.023, 95%CI: 0.724–0.814, *p* < 0.001), TRD + GATA + CTAAAT (AUC 0.824 ± 0.020, 95%CI: 0.784–0.864, *p* < 0.001) and TRD + CTAAAT + ACDG + GATA (AUC 0.81 ± 0.021, 95%CI: 0.765–0.847, *p* < 0.001).

Predictive strengths of susceptibility haplotypes and/or traditional risk factors (TRD) for the diagnosis of osteoporosis were analysed (Figure 3) using AUROC curve by including weighted risk scores (effect estimates). In the first set, four models were used; only TRD, TRD plus haplotype CTAAAT, TRD plus haplotype ACDG and TRD plus haplotype GATA. Area under curve (AUC) revealed marginal and weak analytical power for TRD (AUC = 0.60 ± 0.028, 95%CI: 0.545–0.654, *p* < 0.001), which increased to acceptable limits (AUC = 0.72 ± 0.025, 95%CI: 0.670–0.767, *p* < 0.001) after adding risk scores of haplotype CTAAAT of eNOS gene. Its predictive power marginally increased when TRD were modeled with haplotype ACDG of ACE gene (AUC = 0.614 ± 0.027, 95%CI: 0.560–0.668, *p* < 0.001) whereas, its predictive ability showed maximum strength with haplotype GATA (AUC = 0.8 ± 0.022, 95%CI: 0.751–0.838, *p* < 0.001). In the second set, AUROC was analysed with four different models: (i) TRD + CTAAAT + ACDG, (ii) TRD + ACDG + GATA, (iii) TRD + GATA + CTAAAT and (iv) TRD + CTAAAT + ACDG + GATA, which exposed that third model (TRD + GATA+ CTAAAT) had the highest ability to predict osteoporosis (AUC = 0.824 ± 0.020, 95%CI: 0.784–0.864, *p* < 0.001) followed by fourth model including TRD + CTAAAT + ACDG + GATA (AUC = 0.806 ± 0.021, 95%CI: 0.765–0.847, *p* < 0.001)and TRD + ACDG + GATA (AUC = 0.769 ± 0.023, 95%CI: 0.724–0.814, *p* < 0.001) and with least predictive power of the first model (AUC = 0.713 ± 0.025, 95%CI: 0.665–0.762, *p* < 0.001).

## 4. Discussion

The results obtained in the present study have highlighted that endothelial dysfunction impacts bone mass through genetic participation of *eNOS, ACE* and *VEGFA* genes. Furthermore, this research also illustrates that collaborative effects of genetic variants within these genes along with traditional risk factors are predictive of endothelial dysfunction-affiliated osteoporosis in postmenopausal women. Nonetheless, it is well understood in the clinical arena that hormonal insufficiency in postmenopause phase of life is not only detrimental to bone health, but also influences skeletal vasculature [1,2,3,4] but, whether such connections have genetic connotations, has been largely undefined, primarily because endothelial dysfunction has been considered as a relevant surrogate marker for cardiovascular and cerebrovascular diseases whereas, BMD is used for osteoporosis. In the present analysis, some variants within three noteworthy candidates, which impinge upon endothelial function (*eNOS, ACE* and *VEGFA*), have shown significant impact on the risk of osteoporosis through different genetic models. In individual studies, minor alleles; G, D (deletion) and major allele G of the SNPs of *eNOS*; rs1799983, *ACE*: rs1799752 and *VEGFA:* rs2010963 respectively has been associated with lower BMDs in Chinese Han, Turkish and Caucasian women [30,31,32], which is consistent with the findings of this study.

Whether the influence of these alleles capture the overall genetic variance for the risk of osteoporosis is questionable, as their individual effect may fluctuate when other SNPs also contribute, especially when they are non-randomly linked to each other. Only a few studies have investigated the gene-gene-, gene-environmental- and haplotype-specific effects for osteoporosis risk by involving endothelial function-oriented genes. In a previous report by our laboratory with a lesser sample size (n = 456), a haplotype within eNOS gene CTAAAT has been revealed, bearers of which may have almost double the risk of osteoporosis (OR 2.32, 95% CI: 1.18–4.54, *p* = 0.021) [16]. This inference has been replicated in the present study also by involving larger sample size (n = 676), whereby this susceptibility haplotype exacerbates the risk of osteoporosis after adjusting the effect of confounders (OR 2.43, 95%CI: 1.22–4.71, *p* = 0.007). In both previous and present study this haplotype manifests its risk in multiplicative mode (β ± SE; 2.11 ± 0.63 vs. 2.19 ± 0.86, *p* < 0.05). Nevertheless in order to see the participation and contribution of other pertinent genes (*ACE* and *VEGFA*), results exhibited that two susceptibility haplotypes ACDG (in the order of rs4459609, rs1800764, rs1799752 and rs4343) and GATA (in the order of rs2010963, rs699947, rs833061 and rs1570360) exist within *ACE* and *VEGFA* gene and each unit of these haplotypes increases the osteoporosis risk by 1.73 ± 0.54 and 3.07 ± 0.81 (β ± SE) times respectively, which is manifested in dominant mode.

It is apparently clear that estrogen deficiency, bone loss and estrogen receptor mediated impaired endothelial function are interconnected, which is endorsed by the finding that even estrogen-deficient premenopausal women have higher chances of endothelial dysfunction induced bone loss [33]. Incremental decrease of BMDs at femoral neck and lumbar spine has been observed to be associated with impaired endothelial function in the present study (Figure 2) suggesting that BMD and endothelial dysfunction are linearly related.

It has been shown that SNP rs2070744 is associated with *eNOS* gene expression mediated NO regulation whereby its minor allele C lacks extra transcriptional factors in comparison to major allele T hence, mitigates NO expression in endothelial cells resulting in impaired vascular function [17]. Similarly, minor allele T of another eNOS SNP rs1799983 has also been observed to reduce NO synthesis causing systemic vascular resistance and resulting in impaired vascular function [34]. In *ACE* gene, deletion allele (D) of SNP rs1799752(In/del) regulates about 50 percent of *ACE* levels [21], whereby it impacts vascular function by either superoxide anions or bradykinin induced attenuation of NO. Similarly, minor allele G of SNP rs2010963 within VEGFA gene diminishes VEGF expression [25], consequences of which may impinge upon NO induced bone homeostasis. SNP-SNP interaction analysis have clarified that such association is genetic, which is possibly mediated by epistatic effects between SNP pairs; rs2070744–rs4343, rs4343–rs2010963, rs1799983–rs891512 and rs699947–rs1799752 (Table 3).

It is verified in the present study that individually, neither TRD, nor SNPs are able to capture the overall risk of osteoporosis. Individual SNP-based risk scores have been used to predict osteoporosis [35] but SNPs induce altered influences when traditional risk factors are also present (gene-environment effect) and some SNPs in concert may be epistatic over others. For instance if allele ‘C’ at first position is replaced with allele ‘T’ in susceptibility haplotype CTAAAT considering haplotype TTAAAT, its influence for the risk of osteoporosis diminishes strikingly (from OR 2.43, *p* = 0.007 to OR 1.02, *p* = 0.71). It is also noteworthy that although SNPs rs1800780 and rs3918181 (position 3 and 4 in susceptibility haplotype CTAAAT) are unable to capture any association and are insignificant (*p* > 0.05) in the present analysis (Table 1), but in the presence of other SNPs (CTAAAT position 1, 2, 5 and 6), these SNPs also extend their susceptibility effect, otherwise change in these positions with alleles GG in the haplotype (CTGGAT) would not have diminished association (OR 0.57, *p* = 0.39) for osteoporosis risk (Table 6). Furthermore, it has been confirmed by AUROC analysis in this study that the predictive power of individual TRD is not robust but when its effect is attached to that of susceptibility haplotype within VEGFA gene (GATA), it can predict osteoporosis with substantial power (AUC = 0.8 ± 0.022, *p* < 0.001), which is further supplemented, if, susceptibility haplotype within eNOS gene (CTAAAT) is also included in the analysis (AUC = 0.824 ± 0.020, *p* < 0.001).

## 5. Conclusions

In conclusion, the present study has exposed three susceptible haplotypes (CTAAAT, ACDG and GATA) within eNOS, ACE and VEGFA genes, which manifest in multiplicative and dominant mode for inflicting endothelial-associated osteoporosis risk in postmenopausal women. A model comprising risk scores of traditional risk factors in addition to that of susceptibility haplotype CTAAAT (eNOS gene) and GATA (VEGFA gene) is capable of being excellent predictor of osteoporosis, likelihood of which prompts future genetic studies to probe it.

## Figures and Tables

**Table 1:** Variables at baseline according to osteoporosis status in postmenopausal women having endothelial dysfunction.

Variables	Endothelial Dysfunction		*p* Value
	With Osteoporosis	Without Osteoporosis	
Number of subjects	346	330	-------
Age (years)	60.21 ± 12.4	61.19 ± 8.92	0.934
Years since menopause (years)	8.86 ± 5.03	8.93 ± 3.67	0.106
Body mass index (kg/m^2^)	30.21 ± 3.11	26.2 ± 3.72	<0.001
Systolic blood pressure (mmHg)	129.56 ± 13.72	125.18 ± 12.77	<0.001
Diastolic blood pressure (mmHg)	98.10 ± 9.67	96.11 ± 10.13	0.087
Low density lipoprotein (mg/dL)	199.32 ± 20.18	194.08 ± 19.50	0.441
Triglyceride (mg/dL) ^a^	190(189, 225)	145 (102, 201)	<0.001
High density lipoprotein (mg/dL)	44.23 ± 4.16	45.79 ± 3.15	0.189
Total cholesterol (mg/dL)	227.81± 20.25	221.11 ± 20.78	0.061
BMD_FN(g/cm^2^) ^a^	0.73 ± 0.14	0.89 ± 0.11	<0.001
BMD_LS(g/cm^2^) ^a^	0.84 ± 0.11	0.94 ± 0.14	<0.001
Reactive Hyperemia Index ^b^	1.23 (1.09, 1.34)	1.44 (1.34, 1.55)	<0.001
*eNOS gene/SNPS*			
rs2070744 (MAF ± SE) ^c^	0.26 ± 0.024	0.17 ± 0.015	0.002
rs1799983 (MAF ± SE) ^c^	0.21 ± 0.022	0.10 ± 0.016	<0.001
rs1800780 (MAF ± SE) ^c^	0.43 ± 0.047	0.42 ± 0.027	0.855
rs3918181 (MAF ± SE) ^c^	0.33 ± 0.025	0.29 ± 0.025	0.259
rs891512 (MAF ± SE) ^c^	0.24 ± 0.023	0.16 ± 0.020	0.009
rs1808593 (MAF ± SE) ^c^	0.21 ± 0.022	0.17 ± 0.021	0.189
*ACE gene/SNPS*			
rs4459609(MAF ± SE) ^c^	0.36 ± 0.026	0.35 ± 0.026	0.786
rs1800764(MAF ± SE) ^c^	0.33 ± 0.025	0.26 ± 0.024	0.044
rs1799752(MAF ± SE) ^c^	0.56 ± 0.027	0.46 ± 0.020	0.003
rs4343(MAF ± SE) ^c^	0.31 ± 0.025	0.24 ± 0.023	0.040
*VEGFA gene/SNPS*			
rs2010963(MAF ± SE) ^c^	0.20 ± 0.022	0.27 ± 0.024	0.032
rs833061(MAF ± SE) ^c^	0.42 ± 0.027	0.41 ± 0.027	0.793
rs699947(MAF ± SE) ^c^	0.46 ± 0.027	0.38 ± 0.027	0.036
rs1570360(MAF ± SE) ^c^	0.31 ± 0.025	0.22 ± 0.023	0.008

eNOS: endothelial nitric oxide synthase, VEGFA: vascular endothelial growth factor, ACE: angiotensin converting enzyme, MAF ± SE: minor allele frequency± Standard error, BMD_FN: Bone mineral density at femoral neck and BMD_LS: bone mineral density at lumbar spine. ^a^ Values are corrected with age, body mass index and years since menopause. ^b^ Values are median (25th to 75th interquartile range). All the values are mean ± SD except values ^c^, which are minor allele frequencies ± standard error.

**Table 2:** Regression analyses for the identification of independent risk variables.

Variables	Univariate Analysis			Multivariate Analysis		
	OR	95%CI	*p* Values	OR	95%CI	*p* Values
Body mass index (kg.m^−2^)	1.90	2.10–3.12	**<0.001**	1.45	1.11–2.76	**0.007**
Years since menopause(years)	1.85	2.00–3.89	**0.008**	1.72	1.78–2.52	**0.020**
Diastolic blood pressure (mmHg)	1.61	0.85–2.95	0.281	-----	---------	--------
Systolic blood pressure (mmHg)	1.90	1.33–3.02	**0.009**	1.42	1.12–2.89	**0.012**
Total cholesterol (mg/dL)	1.67	0.91–2.95	0.288	-----	---------	-------
Low density lipoprotein (mg/dL)	2.45	0.87–2.90	0.211	-----	---------	-------
High density lipoprotein (mg/dL)	1.37	0.61–2.35	0.155	-----	---------	------
Triglyceride (mg/dL)	2.31	1.59–3.10	**<0.001**	1.75	1.24–3.09	**0.009**
BMD_FN(g/cm^2^)	1.77	1.28–3.41	**<0.001**	1.20	1.10–2.55	**0.005**
BMD_LS (g/cm^2^)	1.51	1.19–3.23	**0.006**	1.27	1.12–3.00	**0.013**

Categorization in models are; Body mass index (BMI):BMI: <30 vs. ≥30, Years since menopause: ≤5 vs. >5, Diastolic blood pressure: ≤80 vs. >80, Systolic blood pressure: ≤120 vs. >120, Total cholesterol: ≤200 vs. >200, Low density lipoprotein: ≤100 vs. >100, High density lipoproteins: ≥40 vs. <40, Triglyceride: ≤150 vs. >150, BMD_FN: ≥0.7 vs. <0.7, BMD_LS: ≥0.8 vs. <0.8. CI: confidence intervals, BMD_FN (Bone mineral density at femoral neck), BMD_LS (Bone mineral density at lumbar spine). Significant values are shown in bold face.

**Table 3:** Association of *eNOS* gene SNPs for the endothelial dysfunction associated osteoporosis risk.

SNPs/Genetic Model	Input Parameters	Unadjusted OR (95% CI)	*p* Value	Adjusted OR (95% CI)	*p* Value
rs2070744	TT	Referent	-----	Referent	-----
Codominant	TT vs. TC	1.77 (1.26–2.47)	**0.001**	1.55 (1.18–2.19)	**0.007**
Codominant	TT vs. CC	2.15 (1.11–4.15)	**0.032**	1.73 (1.09–3.82)	**0.043**
Dominant	TT vs. TC + CC	1.94 (1.16–3.24)	**0.016**	1.65 (1.10–3.19)	**0.037**
Recessive	TT + TC vs. CC	1.84 (0.67–5.06)	0.343	1.46 (0.59–4.76)	0.287
Multiplicative	2TT + TC vs.TC + 2CC	1.67 (1.28–2.17)	**<0.001**	1.33 (1.14–2.00)	**0.005**
rs1799983	GG	Referent	-----	Referent	-----
Codominant	GG vs. GT	2.86 (1.97–4.15)	**<0.001**	2.53 (1.81–3.91)	**0.006**
Codominant	GG vs. TT	3.18 (1.21–8.33)	**0.025**	3.00 (1.14–8.19)	**0.042**
Dominant	GG vs. GT + TT	3.19 (1.75–5.82)	**<0.001**	2.84 (1.66–5.29)	**0.009**
Recessive	GG + GT vs. TT	2.72 (0.54–13.75)	0.368	2.49 (0.37–10.51)	0.222
Multiplicative	2GG + GT vs. GT + 2TT	2.51 (1.83–3.44)	**<0.001**	2.13 (1.27–2.92)	**0.002**
rs1800780	GG	Referent	-----	Referent	-----
Codominant	GG vs. GA	0.89 (0.64–1.26)	0.580	0.72 (0.59–1.13)	0.491
Codominant	GG vs. AA	1.13 (0.72–1.76)	0.680	1.10 (0.70–1.72)	0.672
Dominant	GG vs. GA + AA	0.91 (0.54–1.53)	0.829	0.81 (0.50–1.48)	0.800
Recessive	GG + GA vs. AA	1.48 (0.79–2.76)	0.280	1.33 (0.71–2.65)	0.271
Multiplicative	2GG + GA vs. GA + 2AA	1.03 (0.83–1.28)	0.890	0.88 (0.71–1.11)	0.786
rs3918181	GG	Referent	-----	Referent	-----
Codominant	GG vs. GA	1.37 (0.99–1.88)	0.067	1.20 (0.82–1.71)	0.079
Codominant	GG vs. AA	1.17 (0.70–1.96)	0.646	1.09 (0.56–1.78)	0.538
Dominant	GG vs. GA + AA	1.16 (0.71–1.89)	0.653	1.00 (0.51–1.70)	0.540
Recessive	GG + GA vs. AA	1.47 (0.68–3.18)	0.423	1.38 (0.62–3.01)	0.411
Multiplicative	2GG + GA vs. GA + 2AA	1.18 (0.94–1.49)	0.177	0.92 (0.69–1.21)	0.165
rs891512	GG	Referent	-----	Referent	-----
Codominant	GG vs. GA	1.68 (1.20–2.34)	**0.003**	1.55 (1.15–2.28)	**0.029**
Codominant	GG vs. AA	2.43 (1.16–5.11)	**0.026**	2.20 (1.10–4.92)	**0.040**
Dominant	GG vs. GA + AA	1.79 (1.06–3.03)	**0.041**	1.67(1.02–2.77)	**0.050**
Recessive	GG + GA vs. AA	1.51 (0.53–4.28)	0.605	1.45 (0.45–4.12)	0.519
Multiplicative	2GG + GA vs. GA + 2AA	1.66 (1.27–2.18)	**<0.001**	1.51 (1.13–2.09)	**0.034**
rs1808593	TT	Referent	-----	Referent	-----
Codominant	TT vs. TG	1.26 (0.85–1.68)	0.330	1.10 (0.71–1.47)	0.290
Codominant	TT vs. GG	1.98 (0.93–4.20)	0.105	1.82 (0.82–4.01)	0.097
Dominant	TT vs. TG + GG	1.57 (0.91–2.69)	0.134	1.43 (0.81–2.37)	0.111
Recessive	TT + TG vs.GG	1.63 (0.53–5.01)	0.558	1.52 (0.41–4.74)	0.419
Multiplicative	2TT + TG vs. TG + 2GG	1.31 (1.00–1.72)	0.061	1.19 (0.87–1.49)	0.091

Assuming genetic disease penetrance of 1, r and r^2^ for genotypes AA, AB and BB respectively, codominant model specifies that risk of osteoporosis is increased by r-fold for genotype AB and r^2^-fold for genotype BB. For dominant model, either one or two copies of allele Bare required for r-fold increased risk, recessive model demonstrates that two copies of allele B are required for r-fold increased risk and multiplicative model indicates that the osteoporosis risk is increased by r-fold with each additional *B* allele. Significant values are shown in bold face.

**Table 4:** Association of *ACE* gene SNPs for the endothelial dysfunction associated osteoporosis risk.

SNPs/Genetic Model	Input Parameters	Unadjusted OR (95%CI)	*p* Value	Adjusted OR (95%CI)	*p* Value
rs4459609	AA	Referent	-----	Referent	-----
Codominant	AA vs. AC	1.09 (0.79–1.51)	0.652	1.00 (0.70–1.45)	0.579
Codominant	AA vs. CC	1.05 (0.64–1.73)	0.944	0.93 (0.55–161)	0.817
Dominant	AA vs. AC + CC	1.08 (0.80–1.40)	0.660	1.00 (0.71–1.26)	0.531
Recessive	AA + AC vs. CC	1.00 (0.63–1.60)	0.920	0.87 (0.58–1.43)	0.779
Multiplicative	2AA + AC vs.AC + 2CC	1.04 (0.89–1.30)	0.749	0.93 (0.76–1.23)	0.654
rs1800764	TT	Referent	-----	Referent	-----
Codominant	TT vs. TC	1.27 (0.92–1.75)	0.167	1.18 (0.85–1.62)	0.141
Codominant	TT vs. CC	1.89 (1.11–3.21)	**0.024**	1.71 (1.08–3.17)	**0.038**
Dominant	TT vs. TC + CC	1.38 (1.02–1.87)	**0.044**	1.21 (1.00–1.73)	0.053
Recessive	TT + TC vs. CC	1.70 (1.02–2.84)	0.053	1.43 (0.92–2.61)	0.092
Multiplicative	2TT + TC vs. TC + 2CC	1.36 (1.08–1.72)	**0.012**	1.22 (1.01–1.62)	**0.043**
rs1799752	II	Referent	-----	Referent	-----
Codominant	II vs. ID	1.57 (1.07–2.31)	**0.027**	1.37(1.05–2.27)	**0.044**
Codominant	II vs. DD	2.28 (1.40–3.56)	**<0.001**	2.19 (1.33–3.41)	**0.007**
Dominant	II vs. ID + DD	1.77 (1.23–2.55)	**0.003**	1.68 (1.20–2.38)	**0.013**
Recessive	II + ID vs. DD	1.66 (1.17–2.37)	**0.006**	1.42 (1.03–2.13)	**0.028**
Multiplicative	2II + ID vs. ID + 2DD	1.48 (1.19–1.83)	**<0.001**	1.22 (1.11–1.69)	**0.008**
rs4343	AA	Referent	-----	Referent	-----
Codominant	AA vs. AG	1.65 (1.19–2.28)	**0.003**	1.51 (1.14–2.17)	**0.009**
Codominant	AA vs. GG	1.63 (0.94–2.85)	0.110	1.49 (0.81–2.13)	0.098
Dominant	AA vs. AG + GG	1.65 (1.23–2.23)	**0.002**	1.43 (1.17–2.00)	**0.028**
Recessive	AA + AG vs. GG	1.33 (0.77–2.28)	0.368	1.24 (0.65–1.99)	0.289
Multiplicative	2AA + AG vs. AG + 2GG	1.44 (1.13–1.84)	**0.003**	1.29 (1.09–1.72)	**0.015**

Assuming genetic disease penetrance of 1, r and r^2^ for genotypes AA, AB and BB respectively, codominant model specifies that risk of osteoporosis is increased by r-fold for genotype AB and r^2^-fold for genotype BB. For dominant model, either one or two copies of allele Bare required for r-fold increased risk, recessive model demonstrates that two copies of allele B are required for r-fold increased risk and multiplicative model indicates that the osteoporosis risk is increased by r-fold with each additional *B* allele. Significant values are shown in bold face.

**Table 5:** Association of *VEGFA* gene SNPs for the endothelial dysfunction associated osteoporosis.

SNPs/Genetic Model	Input Parameters	Unadjusted OR (95%CI)	*p* Value	Adjusted OR (95%CI)	*p* Value
rs2010963	GG	Referent	-----	Referent	-----
Codominant	GG vs. GC	0.63 (0.45–0.87)	**0.007**	0.60 (0.42–0.83)	**0.027**
Codominant	GG vs. CC	0.58 (0.32–1.07)	0.112	0.52 (0.29–1.10)	0.101
Dominant	GG vs. GC +CC	0.62 (0.46–0.85)	**0.003**	0.57 (0.41–0.79)	**0.036**
Recessive	GG +GC vs. CC	0.69 (0.38–1.25)	0.282	0.63 (0.29–1.10)	0.178
Multiplicative	2GG + GC vs. GC + 2CC	0.68 (0.53–0.88)	**0.004**	0.65 (0.50–0.82)	**0.010**
rs833061	CC	Referent	-----	Referent	-----
Codominant	CC vs. CA	0.97 (0.69–1.35)	0.905	0.84 (0.55–1.22)	0.811
Codominant	CC vs. AA	1.12 (0.73–1.73)	0.674	1.05 (0.63–1.65)	0.555
Dominant	CC vs. CA + CC	1.01 (0.74–1.38)	0.978	0.89 (0.63–1.19)	0.818
Recessive	CC +CA vs. AA	1.15 (0.78–1.69)	0.550	1.12 (0.72–1.63)	0.532
Multiplicative	2CC + CA vs. CA + 2AA	1.05 (0.84–1.30)	0.713	0.92 (0.76–1.18)	0.582
rs699947	TT	Referent	-----	Referent	-----
Codominant	TT vs. TC	1.29 (0.92–1.82)	0.161	1.18 (0.80–1.65)	0.145
Codominant	TT vs. CC	1.86 (1.20–2.88)	**0.008**	1.72 (1.11–2.72)	**0.027**
Dominant	TT vs. TC + CC	1.43 (1.04–1.97)	**0.032**	1.30 (1.00–1.82)	**0.048**
Recessive	TT +TC vs. CC	1.60 (1.08–2.38)	**0.024**	1.51 (1.01–2.24)	**0.039**
Multiplicative	2TT + TC vs. TC + 2CC	1.37 (1.10–1.70)	**0.005**	1.22 (1.05–1.61)	**0.027**
rs1570360	GG	Referent	-----	Referent	-----
Codominant	GG vs. GA	1.64 (1.18–2.27)	**0.004**	1.52 (1.11–2.19)	**0.011**
Codominant	GG vs. AA	2.26 (1.28–3.99)	**0.006**	2.13 (1.17–3.29)	**0.026**
Dominant	GG vs. GA + AA	1.74 (1.28–2.37)	**<0.001**	1.69 (1.20–2.31)	**0.017**
Recessive	GG +GA vs. AA	1.87 (1.07–3.25)	**0.035**	1.74 (0.92–2.88)	0.062
Multiplicative	2GG + GA vs. GA + 2AA	1.62 (1.27–2.07)	**<0.001**	1.53 (1.18–1.95)	**0.025**

Assuming genetic disease penetrance of 1, r and r^2^ for genotypes AA, AB and BB respectively, codominant model specifies that risk of osteoporosis is increased by r-fold for genotype AB and r^2^-fold for genotype BB. For dominant model, either one or two copies of allele Bare required for r-fold increased risk, recessive model demonstrates that two copies of allele B are required for r-fold increased risk and multiplicative model indicates that the osteoporosis risk is increased by r-fold with each additional *B* allele. Significant values are shown in bold face.

**Table 6:** Significant SNP-SNP cross talks amongst *eNOS, ACE* and *VEGFA* genes and their association with traits.

**SNP**	**SNP**	**Trait**	**Test**	***P*^O^**	***P*^NO^**	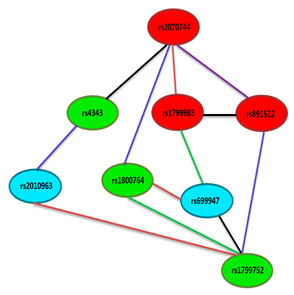
rs2070744	rs4343	RHI	I	0.0013	0.432	
rs2070744	rs1799983	LDL	AA	0.0052	1.348	
rs2070744	rs1800764	TG	DA	0.0011	0.041	
rs2070744	rs891512	SBP	AD	0.0035	0.122	
rs4343	rs2010963	RHI	AA	0.0090	0.178	
rs1800764	rs1799752	TC	DD	0.0290	2.152	
rs1799983	rs891512	RHI	I	0.0320	1.813	
rs1799983	rs699947	BMI	DD	0.0014	0.041	
rs699947	rs1799752	RHI	I	0.0035	0.082	
rs891512	rs1799752	LDL	DA	0.0221	0.078	

Many SNP-SNP interactions were deduced between groups of women with osteoporosis and women without osteoporosis, but only significant effects are shown here. All *p* values are Bonferroni corrected. *P*^O^: *p* values showing gene-gene effect (SNP × SNP) influencing risk variables in women with osteoporosis (n = 346), *P*^NO^: *p* values showing gene-gene effect (SNP × SNP) influencing risk variables in women without osteoporosis (n = 330). Two-locus effects of these SNP pairs indicate AA: additive x additive (Red line color), AD: additive x dominant (Purple line color), DA: dominant x additive (Blue line color), DD: dominant x dominant (Green line color) and I: interactive effect (Black line color). Color of the ellipse indicates Red: eNOS gene, Green: ACE gene and Cyan: VEGFA gene. RHI: reactive hyperemia index, LDL: low density lipoprotein, TG: triglyceride, SBP: Systolic blood pressure, TC: total cholesterol and BMI: body mass index.

**Table 7:** Haplotypes within eNOS, ACE and VEGFA genes and their influence on osteoporosis risk.

Haplotype	Endothelial Dysfunction		P*_Cor._*	Unadjusted OR (95%CI)	*p* Value	Adjusted OR(95% CI) ^a^	*p* Value
	With	Without					
	Osteoporosis	Osteoporosis					
***eNOS gene***							
TGAAGT	0.21 (73)	0.20 (66)	0.97	Referent	--------	Referent	------
TTAGGG	0.11 (38)	0.15 (49)	0.21	0.70 (0.41–1.20)	0.25	0.61 (0.32–1.13)	0.19
TTGGGG	0.09 (31)	0.12 (40)	0.31	0.70 (0.39–1.25)	0.28	0.52 (0.32–1.19)	0.22
CGAAGG	0.10 (35)	0.12 (40)	0.71	0.79 (0.45–1.39)	0.50	0.72 (0.33–1.21)	0.39
**CTAAAT**	**0.18(62)**	**0.06 (20)**	**1 × 10^−8^**	**2.80 (1.53–5.13)**	**0.001**	**2.43 (1.22–4.71)**	**0.007**
CGGAGG	0.07 (24)	0.08 (26)	0.93	0.83 (0.44–1.59)	0.72	0.65 (0.39–1.42)	0.50
CTGGAT	0.06 (21)	0.08 (26)	0.63	0.73 (0.38–1.42)	0.45	0.57 (0.34–1.21)	0.39
TTAAAT	0.06 (21)	0.05 (16)	0.80	1.19 (0.57–2.46)	0.78	1.02 (0.48–2.35)	0.71
***ACE gene***							
ATIA	0.31 (107)	0.35 (115)	0.50	Referent	--------	Referent	------
**ACDG**	**0.27 (93)**	**0.10 (33)**	**1 × 10^−9^**	**3.03 (1.86–4.88)**	**<0.001**	**2.50 (1.28–3.96)**	**0.002**
CTIG	0.12 (41)	0.14 (46)	0.72	0.96 (0.58–1.57)	0.97	0.80 (0.42–1.21)	0.42
CCIG	0.11 (38)	0.12 (40)	0.93	1.02 (0.61–1.71)	0.96	0.98 (0.33–1.38)	0.71
ACDA	0.09 (31)	0.11 (36)	0.70	0.93 (0.54–1.60)	0.89	0.68 (0.47–1.16)	0.67
***VEGFA gene***							
GCTG	0.28 (86)	0.29 (96)	0.38	Referent	--------	Referent	------
**GATA**	**0.19 (66)**	**0.08 (26)**	**1 × 10^−7^**	**2.83 (1.65–4.86)**	**<0.001**	**2.10 (1.31–3.29)**	**0.009**
GACG	0.14 (48)	0.16 (53)	0.73	1.01 (0.62–1.65)	0.94	0.92 (0.42–1.19)	0.65
GCTA	0.12 (41)	0.13 (43)	0.93	1.06 (0.63–1.79)	0.92	0.87 (0.41–1.11)	0.60
**CACG**	**0.06 (21)**	**0.14 (46)**	**<0.001**	**0.51 (0.28–0.92)**	**0.035**	0.78 (0.45–1.33)	0.43
CCTG	0.09 (31)	0.11 (36)	0.70	0.96 (0.55–1.69)	1.00	1.05 (0.61–1.83)	0.96

Number of subjects having haplotype are shown in the parenthesis. All those haplotypes which had less than 5 percent frequencies (eNOS gene: 21, ACE gene: 6 and VEGFA: 5) were excluded from the analysis. P*^Cor^*-*p* values are corrected for multiple comparisons (Bonferroni adjustment). Bold faces show the susceptibility haplotype. ^a^ Odds ratios are adjusted with body mass index, years since menopause, systolic blood pressure, triglyceride levels. Significant values are shown in bold face.

**Table 8:** Functional implications of susceptibility haplotypes and their impact in best fit model.

eNOS-Haplotype CTAAAT					
Model	^a^ β ± SE	Wald Test	*p* Value	*R* ^2^_h_	AIC
Dominant	0.33 ± 0.43	0.77	0.440	0.6805	5346.49
Recessive	0.22 ± 0.29	0.76	0.291	0.6192	5892.21
**Multiplicative**	**2.19 ± 0.86**	**2.55**	**<0.001**	**1.000**	**3336.28**
General (0 copy)	−0.30 ± 0.39	−0.77	0.440	0.9790	5342.96
General (1 copy)	2.10 ± 0.82	2.57	0.010	0.9790	5342.96
ACE-Haplotype ACDG					
**Dominant**	**1.73 ± 0.54**	**3.19**	**0.001**	**1.000**	**1076.05**
Recessive	0.43± 0.89	0.48	0.626	0.896	1298.44
Multiplicative	0.10 ± 0.36	0.29	0.769	0.916	1082.75
General (0 copy)	−0.88± 0.32	−2.70	0.006	0.891	1090.10
General (1 copy)	−0.05± 0.41	−0.12	0.898	0.891	1090.10
VEGFA-Haplotype GATA					
**Dominant**	**3.07 ± 0.81**	**3.79**	**<0.001**	**1.000**	**5324.21**
Recessive	0.36 ± 0.43	0.84	0.399	0.680	5337.60
Multiplicative	0.59 ± 0.48	1.23	0.216	0.722	5335.36
General (0 copy)	−0.58 ± 0.45	−1.28	0.198	0.935	5621.86
General (1 copy)	0.004 ± 0.14	0.028	0.977	0.935	5621.86

Models showing values after adjustment for risk covariates; body mass index, years since menopause, systolic blood pressure, triglyceride levels and BMDs at femoral neck and lumbar spine. ^a^ Estimated haplotype effect, P-asymptotic value, *R*^2^h-haplotype uncertainty measure, AIC- Akaike information criterion. Values in bold face show highest *R*^2^_h_ values and lowest AIC. Dominant effect (women having one copy is at same risk as those women having two copies), Recessive (women having one copy is at the same risk as women having no copy), Multiplicative effect (women having one copy of the haplotype are at intermediate risk than women having no copy or two copies). Significant values are shown in bold face.

## Data Availability

The data presented in this study are available on request from the corresponding author. The data are not publicly available due to ethical issues.
